# Patient‐initiated recruitment for clinical research: Evaluation of an outpatient letter research statement

**DOI:** 10.1111/hex.12642

**Published:** 2017-11-22

**Authors:** Matthias Wienroth, Louise Caffrey, Charles Wolfe, Christopher McKevitt

**Affiliations:** ^1^ School of Population Health & Environmental Sciences Faculty of Life Sciences and Medicine King's College London Guy's Campus London UK; ^2^ Policy, Ethics and Life Sciences Research Centre Newcastle University Newcastle upon Tyne UK; ^3^ School of Social Work and Social Policy College Green Trinity College Dublin Dublin 2 Ireland; ^4^ NIHR Biomedical Research Centre at Guy’s and St. Thomas’ NHS Foundation Trust and King’s College London London UK

**Keywords:** clinical research, patient recruitment, research literacy

## Abstract

**Background:**

UK Hospital Trusts are charged with increasing patients’ research awareness and willingness to take part in research. This includes implementing strategies to encourage patient‐initiated enquiries about participation.

**Objectives:**

To evaluate the impact of a research statement inserted in outpatient letters in one clinical service, and to derive suggestions on potential steps towards increasing patient‐initiated recruitment.

**Setting:**

A medical outpatient clinic of a research‐active hospital trust, serving an inner‐city multi‐ethnic population across two boroughs.

**Methods:**

Pre‐intervention and post‐intervention questionnaires were administered face‐to‐face to new patients. Questionnaires included closed questions and one open comments section. Data were analysed for frequencies, with thematic coding of open‐ended responses.

**Results:**

The response rates were 87% for the pre‐intervention survey and 92% for the post‐intervention survey. In the post‐intervention survey, 85% of patients did not notice the research statement in the letter. More than half found the statement “a little unclear,” whilst one‐third considered it “clear.” Three‐quarters of respondents perceived the statement to be “a little helpful.” Only one person enquired about participating in clinical research having read the statement in the outpatient letter.

**Conclusion:**

The analysis suggests that simple, single‐solution approaches such as including research statements in outpatient letters are unlikely to be sufficient to significantly facilitate patient‐initiated recruitment. Recruitment efforts need to take into consideration the diversity of patient constituencies including the reasons they seek health care, and how patients can meaningfully access information (research literacy).

## 1 INTRODUCTION

Since the 1990s, a greater role of patients in clinical research in the United Kingdom (UK) has been advocated, with patients positioned as “consumers” and “service users,” rather than as “research subjects.”^1^, ^2^ At the same time, the UK's National Institute of Health Research (NIHR) has sought to encourage greater participation of patients in clinical research conducted in the National Health Service (NHS). Long‐standing concerns about shortcomings in participant recruitment to clinical trials and other types of health research have been widely reported. A Cochrane review that included 45 trials, reported that “it is likely that less than 50% [of clinical studies] meet their target [in patient recruitment], or meet their target without extending the length of the trial.”^3^ The authors suggest that recruitment may be viewed as problematic by clinical research stakeholders, outlining three main concerns: At the scientific level, “underpowered” studies may report as statistically non‐significant results which are nonetheless clinically relevant; ethically, participants in “underpowered” studies have been exposed to interventions with uncertain benefit but the effectiveness of the intervention remains uncertain; and on economic grounds, they underline that greater costs may be incurred if “underpowered” studies need to be extended to arrive at statistically significant results. These three concerns provide a framework within which to understand why stakeholders consider greater numbers of participants as vital to the efficiency and efficacy of clinical research .

Patient recruitment into clinical research is subject to three core considerations: approach (how to reach sufficient numbers of participants), eligibility and retention. To increase recruitment, the NIHR has proposed that UK health organizations develop a “research culture,” focused on delivering innovation in research and care provision,^4^ which encourages patients to proactively seek involvement in clinical research. The clinical research network aims to support the development of such a “research culture” by “providing NHS Trusts with additional funding to cover the cost of research nurses and other clinical research delivery staff, who identify and approach patients about relevant research opportunities.”^5^ Not only does this work aim at recruiting patients, but even more so at encouraging patients to voluntarily and of their own accord come forward to enquire about, and subsequently participate in clinical research. However, the NIHR's mystery shopper campaign conducted at 82 hospital sites in England in 2012, found that very little information on clinical research is made available to patients at point of care,^5^ evidencing concern about difficulties with recruitment to NHS research. Subsequently, the NIHR devised an annual patient engagement campaign, until recently called “OK to Ask” (https://www.nihr.ac.uk/patients-and-public/documents/OK-to-ask-report.pdf), and since 2017 entitled “I am Research” (https://www.nihr.ac.uk/news-and-events/support-our-campaigns/i-am-research/), to raise awareness of opportunities for patient participation in research. Such efforts are premised on expectations that patient‐initiated recruitment can significantly increase participation numbers and reach also those patients that may not be included in hospital‐based patient recruitment drives. The NIHR's “OK to ask” campaign (2013‐16) had positioned patient participation in clinical trials as a right, and the campaign was framed in terms of empowerment to ask about clinical research and potential participation in it. The recent shift to “I am Research” frames research participation as both a right and an obligation, a way to “give back” to the health‐care system to help improve care whilst benefitting on various levels including generating a sense of community, becoming more informed about illness, gaining access to latest treatments and gaining a feeling of more control over their illness. The messages of “I am Research” dually frame patient participation in terms of reciprocity for health‐care provision based on equity or solidarity whilst calling upon the imagined patient's more economic reasoning of cost‐benefit.

## 2 CASE STUDY

This case study took place in one medical outpatient department of an academic hospital trust, serving two inner‐city two boroughs, tertiary referrals from a larger geographical area. The local population is characterized by high levels of ethnic diversity: in one borough, Black Africans and Caribbeans make up one‐quarter of the population, whilst in the other, this proportion is 31% of the total. There are wide variations in socio‐economic status, with these boroughs ranking as the 22nd and 41st most deprived in England. The Trust is highly research active, consistently ranking very highly in NIHR research league tables in terms of studies and patients recruited to research. It is also active in promoting research to patients, taking part in annual Clinical Trials information days, including information about research on its website and in its patient and public engagement strategy, and supporting a number of condition‐specific patient and public involvement (in research) groups.

The Trust responded positively to the “OK to ask” campaign's recommendation to include a statement on research in each patient admission letter and one clinical department agreed to pilot the statement in letters for outpatients attending a medical clinic for the first time following referral from a general practitioner. We have previously reported on this process, highlight the resource intensive nature of its implementation.^6^ The statement wording was devised by Trust staff and had considerable input from lay members with experience of patient and public involvement and of research participation. The purpose of the statement was to raise patient awareness of the Trust as, not only as a health‐care provider, but also a research centre, and to encourage patients to ask about research, with a view to increasing patient‐initiated recruitment. The final wording was adopted as follows:

Our hospitals are involved in developing new treatments and better care. If you would like to take part in a research study or want to know more about taking part, please speak to the doctor or nurse caring for you. If you are asked to take part in a research study, we will explain it to you in detail. If you decide not to take part, this will not affect your treatment in any way.

On 24 October 2014, the piloting of the initiative began when the statement above was inserted into the outpatient letter template for patients attending their first appointment (Supplementary Appendix S1). This paper analyses findings of an evaluation of this pilot scheme, which aimed to understand if and to what degree the research statement in the admission letter impacted on patient awareness of, and patient‐initiated recruitment to, the research activities of the Trust. The analysis focuses on patient perceptions of the pilot statement and discusses outcomes and implications of the pilot test for facilitating patient‐driven recruitment to clinical research. The paper then discusses these findings within the context of the NIHR's patient engagement and recruitment.

## 3 METHODS

**Figure 1:**
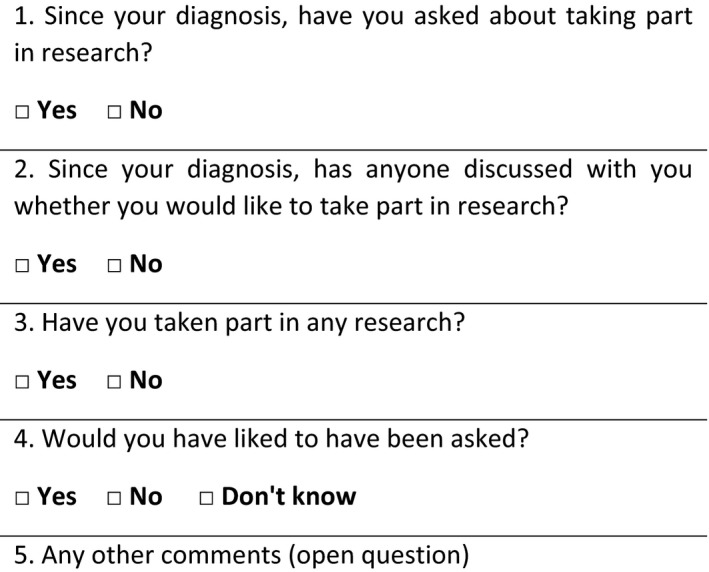
Pre‐intervention survey

**Figure 2:**
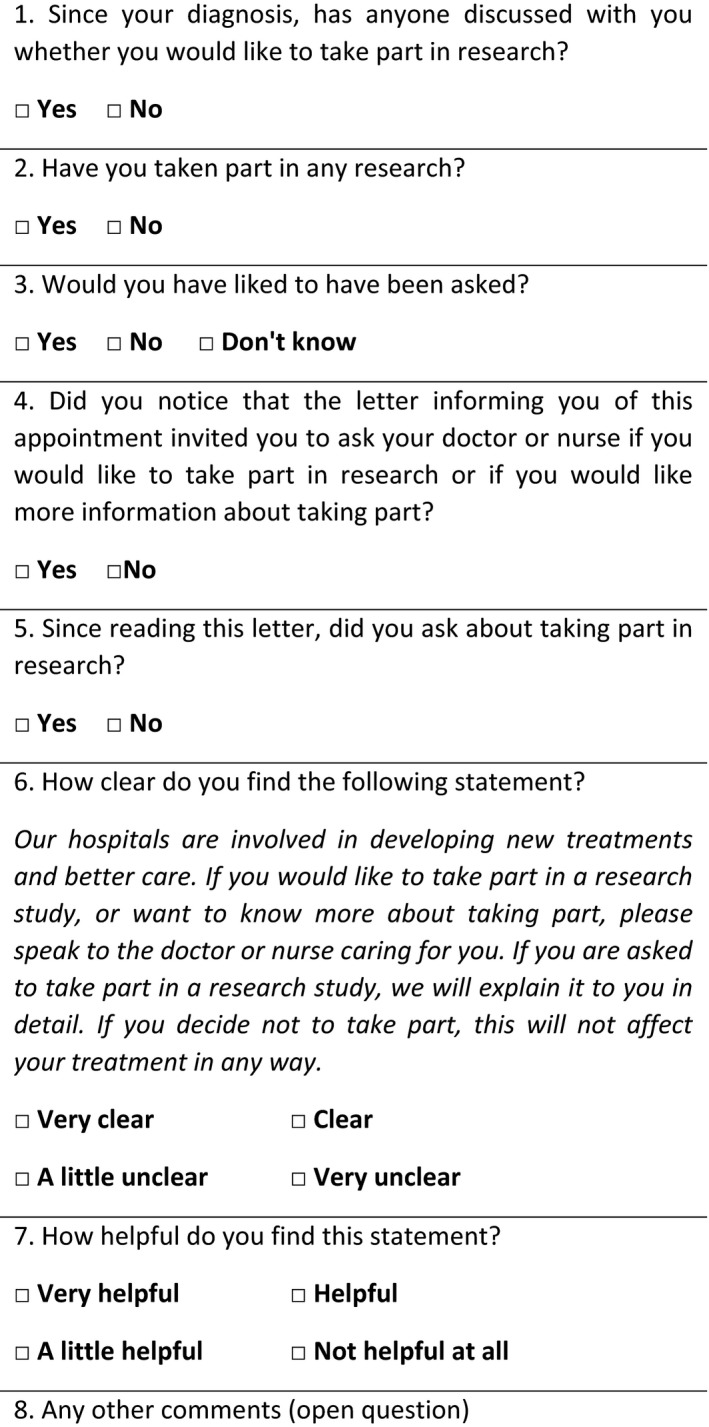
Post‐intervention survey

Pre‐ and post‐intervention surveys were administered to different groups of outpatients in one clinic immediately following their first appointment. The questionnaires included primarily closed questions, and one open question, aimed at contextualizing responses. The questions used are shown in Figures 1 and 2.

The closed questions in the pre‐intervention questionnaire were taken from an unpublished 2013 survey in the same clinics, which in turn had been adapted from validated questions used in the 2011/2012 National Cancer Patient Experience Survey.^7^ It was intended that the pre‐intervention survey would provide a baseline comparison of the number of patients asking about research prior to the introduction of the letter statement. However, it became apparent in the evaluation process that the question “Since your diagnosis, have you asked about taking part in research?” was not suited to assessing patient enquiries about research in this case because patients had been referred to the clinic by their general practitioner (GP) to receive a confirmed diagnosis. Therefore, answers to this question do not provide a clear indication of the frequency of patients asking about research in the Trust. The question was amended in the post‐intervention survey to specifically ask “Since reading this letter, did you ask about taking part in research?”

### 3.1 Delivery of the questionnaire

Research assistants conducted the survey face‐to‐face, recording participants’ responses via the website https://www.surveymonkey.com/ on a hand‐held tablet device. All new patients to the outpatient clinic, identified by clinic staff, asked to participate in this evaluation. Participants were approached immediately after their medical appointment to provide an opportunity for them to have asked about taking part in clinical research should they so wish.

The pre‐intervention survey was administered at seven clinics between late November and early December 2014. The post‐intervention survey was conducted at seven clinics between the end of February and the beginning of March 2015. The pre‐intervention survey took around 3 minutes to administer, the post‐intervention survey around 5 minutes. Both were deliberately short to encourage a high response rate. No identifying patient data were collected. Ethics committee approval was not required as the survey was NHS pre‐assessed to fall within the category of “service evaluation” (http://www.hra.nhs.uk/research-community/before-you-apply/determine-whether-your-study-is-research/).

### 3.2 Data analysis

Survey responses were tabulated and analysed for frequencies. Open‐ended comments were recorded verbatim and coded to facilitate grouping into broader categories. At this stage, findings were presented to two local patient and public involvement groups, with discussion on interpretation of the results and their implications.

### 3.3 Survey results

In total, 453 patients were approached to take part in the two surveys, of whom 405 participated, resulting in an overall response rate of 90%. The pre‐intervention survey produced 207 responses (87% of 237 approached), and the post‐intervention survey 198 responses (92% of 216 approached).

#### 3.3.1 Impact of the research statement

This section focuses on results from the survey designed to assess the impact of the statement (post‐intervention).

Table 1 summarizes key closed questions from the post‐intervention survey in relation to patient responses to the letter statement. A more detailed presentation of the results follows, including data from the open questions.

**Table 1:** Participant reactions to the letter statement

Notice the statement (Q4) (%)	
Yes	15
No	85
Clarity of the statement (Q6) (%)	
Very clear	4
Clear	33
A little unclear	56
Very unclear	7
Helpfulness of the statement (Q7) (%)	
Very helpful	1
Helpful	10
A little helpful	76
Not helpful at all	13

#### 3.3.2 Notice

Only 15% of participants indicated that they noticed the letter statement; 85% said they did not. In the Open Comments section of the survey, patients suggested that they did not notice the statement because they had used the letter to primarily learn about the time and date of their appointment, or because they had lost the letter or disposed of it once they had noted the details for their appointment.

#### 3.3.3 Clarity

When participants read the statement, the majority (56%) found its content “a little unclear”, whilst 7% perceived it to be “very unclear”. A third (33%) of patients felt that the statement was “clear,” and only a minority of 4% found it “very clear”. In their Open Comments, two patients expressed difficulty understanding the statement, suggesting, respectively, that it was “too wordy” and “too long”. Many participants noted that the statement did not sufficiently explain the concept of research. Even when patients agreed that the wording of the statement was clear, some indicated they did not have sufficient information about the idea of research per se to enable them to actively ask about becoming involved. Lack of clear communication was explicitly indicated as the reason why some patients reported insufficient motivation to ask about clinical research, with some stating that their potential role in research was not clearly described and because of this the statement did not encourage them to ask about research.

#### 3.3.4 Helpfulness

The statement was considered to be of “little help” for encouraging research participation by 76% of participants, only a minority found it helpful. Some participants reported through the Open Comments that they had noticed the statement and were interested in taking part, but had not asked about research because they forgot to ask, or they were concerned they would “waste” time in their clinical appointment, which they felt was already short.

The pre‐intervention survey provides baseline data on the propensity of patients in this clinic to ask about taking part in research. Thirteen per cent (n = 26, of 207) reported that they had asked about taking part in research. However, this response includes the enquiries patients made about research to any health‐care professional, including those outside the Trust, and is not limited to enquiries made during patients’ first appointments at the Trust.

Patients participating in both surveys were asked if, since their diagnosis, anyone had discussed with them whether they would like to take part in research. Of the 321 patients (70% of the overall sample of approached patients in both surveys) who had never been approached to take part in clinical research, 43% responded that they would not like to have been asked. Meanwhile, 24% said they would like to have been asked, and 33% said they did not know whether or not they would have like to have been asked. These findings suggest that the health‐care integrated partnership between clinical and public stakeholders for research is not as developed as NIHR ambitions would like them to be. The open comments provided some further information about why patients were not interested in research, including that some patients felt nervous or mistrustful of research; some felt that the seriousness of their illness did not merit participation in research (not sufficiently serious) or their diagnosis and treatment pathway were clear; and some participants considered a clinical appointment an inappropriate occasion to discuss research as they perceived there would be a conflict of interest if their clinician were to discuss research participation with them. Nevertheless, there is a considerable constituency of patients that would be ready to engage in discussions about research even if they were not inspired by the research statement to initiate an enquiry.

The finding that only one patient of 195 asked about taking part in research after reading the research statement suggests that, in this pilot intervention, a research statement inserted into an outpatient letter did not have a large immediate effect on patient‐initiated recruitment.

### 3.4 Limitations of the evaluation

It is important to note the limitations of the evaluation. First, whilst few patients asked about research at their first appointment, they may yet ask at later appointments. In this sense, the evaluation can only provide an early snapshot of the impact of the letter statement. Second, the letter statement may have greater impact in a different context with patients who have fewer treatment options and so may be more interested in experimental treatments. However, a focus on personal gain for therapeutic interventions from research is not the only driver for patient involvement in research.^8^, ^9^, ^10^, ^11^ Third, the difference of questions—asking about taking part in research—between pre‐ and post‐intervention surveys translates into limited currency for comparison of baseline with post‐intervention data to test for significant differences. However, the change in wording offered a more specific indication of the level of patient enquiry about research after the letter was introduced and provided a useful indication of the immediate impact of the statement.

## 4 DISCUSSION

This evaluation found that a small proportion of patients (13%) had previously enquired about research participation, although we do not know where or when this enquiry had been made. We also found the research statement had limited success encouraging patients to ask about participating in clinical research (1 of 195 participants). There are several possible reasons for this: the “visibility” of the statement, being of black text at the foot of the letter; the possibility that patients were primarily concerned about the information related to their first specialist appointment; or their prioritizing getting a confirmed diagnosis from a hospital specialist. A large majority (70%) reported never being asked to take part in research; of these, 43% would not like to have been asked and 33% did not know whether or not they would have liked to have been asked. These results should be compared with other studies investigating patients’ willingness to be informed about and participate in research, where reported estimates vary, depending on setting and population. In a 2000 study of a random population sample in the United States, 46% reported being willing to take part research on new treatments for a disease that was of concern to them, with 29% undecided.^8^ A Korean survey found that 25% of randomly selected members of the public would be willing to participate in a future trial,^9^ with the same proportion reported from a German study of the general public.^10^ Patient populations report higher levels of willingness to participate. In a 2006 survey of 400 outpatients at a general internal medicine practice at a tertiary care academic medical centre in the United States, 68% showed “interest” in participating in clinical trials.^11^ The UK National Cancer Experience Survey reported that 95% of patients who had research discussed with them were happy to have been asked and 53% of those with whom research was not discussed would have been happy to have been asked.^7^ Moorcraft et al^12^ report that most patients in a specialist cancer hospital were happy to be approached about research participation, and 88 % of those approached during the study period consented to take part in a clinical trial.^12^ However, they note that “patients who have just started their first treatment for cancer are less likely to participate in cancer research and it appears that as time increases from diagnosis, patients are more positive about engaging with research” (2016:8).

The Cochrane review by Treweek and colleagues,^3^ cited in the Introduction to this paper, seeks to quantify the effects of strategies to improve recruitment of participants to randomized controlled trials. They report that promising approaches include telephone reminders; requiring potential participants to opt‐out of being contacted by the trial team regarding taking part in a trial, rather than them having to opt‐in; and open designs. These recruitment strategies presuppose prior identification and access to patients who represent eligible potential trial participants and require considerable time investment by the trial team who will also be busy fulfilling other roles in the research process. As such, it is likely that such strategies can only meaningfully increase patient participation with groups that are easy to access by trial teams. Recent and ongoing efforts by the NIHR (eg, the two campaigns and the research statement in patient admission letters), however, aim to reduce the effort necessary to recruit by shifting work to patients themselves, and to broaden the constituency of patients participating in trials. The research statement may have been deployed expecting patients to have been engaged already by the “OK to Ask” campaign, which aims to make the idea of clinical research accessible to patients, and to emphasize those aspects reported as enablers in the literature. There is little evidence of the effectiveness of this campaign but the results of this evaluation suggest that the research statement of itself is not effective, at least for new clinic attenders.

The exploration of diverse barriers and enablers to public participation in clinical research has become a vital resource for understanding existing recruitment limitations and provides a basis for developing proposals on how to address these.^13^ This literature shows that patients have very diverse and also multiple reasons for either participating or not participating in research.^14^, ^15^, ^16^, ^17^ A closely related body of scholarship explores the under‐representation of certain groups of patients in research, reflecting underlying concerns about equity and equality, and about the impact of under‐representation of specific groups on the validity of the research. Recurring categories here include (but not limited to) ethnicity,^18^ language skills,^19^ age,^20^ socio‐economic status,^21^ educational levels^22^ and awareness of research and research literacy.^23^, ^24^ This body of work shows that potential trial participants come from very diverse constituencies and that there are both structural (social and research institutional) as well as personal barriers to participation. These efforts, at least in part, can provide the means to compensate for an increasing methodological formalism in recruiting patients which threatens to substitute substantive concern with the reasons why certain populations may be hard to recruit into clinical research.^25^ Such formalism can be seen in the inclusion of the generic statement on clinical research in patient admission letters. The research statement presupposes an idea of patients as a homogeneous group, who already have access to information about clinical studies, who sufficiently understand the content of the statement as well as the aims and methods of research (ie, research literacy), and who would, therefore, be sufficiently convinced to proactively pursue participation in trials.

For patients in this case study, however, very specific circumstances come to bear. That such differences can have a material impact on the engagement with material such as a research statement may not have been considered when the intervention was developed. The participants of this survey attended the appointment with a hospital specialist to receive confirmation of diagnosis and if necessary to initiate clinical treatment. Medical sociology discusses “diagnosis” in terms of product and enabler of social actions,^26^, ^27^ as a “medical reading” of symptoms that a patient presents with; the concept describes a social process in which physician and patient negotiate symptoms and their meanings. Patients may associate clarification, the framing of their health situation, with diagnosis, which can help them make sense of concerns about, and material impacts from disease. As such, it can help patients feel like they are managing, or taking some control over their health condition,^28^ turning the biomedical disease into an embodied, experienced illness.^29^ When prioritizing diagnosis as their rationale for engaging with the health‐care system, as in the case study, patients expect the health‐care provider to be adequately prepared to help them manage their health condition or provide therapy towards overcoming the experience of illness. Patients may not even associate health‐care provision with research as they expect their physician to already possess the knowledge for treatment. Such an expectation would make a request for participation in research seem irrelevant, or perhaps even unsettling to the patient, if it is not further detailed and made accessible in such a way that the statement encapsulates the specific situation of the person addressed by it.

Similarly, the research statement in the letter is aimed at an audience. This audience is constituted by a specific public made up of patients who are encouraged to volunteer for clinical research by informing themselves about research and trials currently under way, to subsequently come forward to register on a research database, independently of health‐care professionals asking them to participate in a specific trial. Warner reminds us that audiences are created in the process of addressing them^30^ that publics stand in relation to an issue and an addresser. The research statement in the letter aims to address not just any public but an “active,” “attentive” public who understands the context and rationale for the statement to be able to act upon it and has an interest in doing so. To be attentive, the patient audience needs to “have a special incentive for assessing the common content of issues, for they have a particular stake in issues… have greater knowledge about the issue domain and should be more aware of the implications of issues.”^31^ This cannot be presupposed for as diverse a public as health‐care service users, especially not when taking into consideration the empirical findings of research on barriers to research and participation, and on under‐represented groups, each of which have a variety of characteristics defining specific constituencies and helping to understand their limitations in accessing research. The statement in the letter does not address each constituency in terms capable of overcoming such limitations.

The findings from this case study lead us to suggest that perhaps the UK health‐care system—particularly the relationship between care provision and clinical research—remains black‐boxed to a large part of the patient population and does not reproduce the attentive audience imagined by the NIHR and its institutional executors as potential research participants. To be able to reach as many different patients as possible and to “activate” these towards patient‐initiated recruitment, the broad concept of patient may need to be disaggregated so that specific audiences can be addressed.

## 5 CONCLUSION

Our results suggest that simple, single‐solution approaches to increasing research awareness—such as including research statements in outpatient letters—are unlikely to be sufficient to significantly facilitate patient‐initiated recruitment for clinical research, even in the context of a research‐active clinical organization, already employing other strategies to promote research awareness and participation. There is a need for further research into how to identify and approach different patient constituencies appropriately for research. To do so, a synthesis of various forms of extant empirical research and scholarship as well as engagement methods are needed, including on barriers and enablers of research and participation; on reasons for which certain groups are under‐represented; on the conceptualization of illness, diagnosis, patient and (clinical) research; on community‐based work;^22^ on identifying needs for improving research literacy;^23^, ^24^ and on the embedding of research with and for society, as proposed for science by the responsible research and innovation (RRI) programme of the European Union.^32^

Therefore, there is an ongoing need to develop the health‐care system's approach to patients: patients are not one homogenous public, but are diverse publics that need to be addressed as specific audiences along a broad continuum of different priorities and needs, values and perceptions.

## CONFLICT OF INTEREST

The authors declare no conflict of interest.
