# Supplementary material for: Patient‐initiated recruitment for clinical research: Evaluation of an outpatient letter research statement
Source: Health Expect. 2017 Nov 22;21(2):494–500. doi: 10.1111/hex.12642 (PMC5867329; doi:10.1111/hex.12642)
Supplement: Supplementary file 1 [file HEX-21-494-s001.docx]

**Outpatient appointment with *(specialist)* clinic.**

We have booked the earliest available appointment for you to attend a clinic at **Name Hospital**.

Appointment details:

**Date and time:               XXXX**

**Clinic:                            (Specialist Clinic location)**

**Address:                        (Details of location)**

**Under the care of:        (Specialist Team Descriptor)**

**IMPORTANT: Please bring a list of all your current medication with you to this appointment.**

If this appointment is inconvenient and you need to change it, please call us on (**telephone number) or e-mail (**[emailaddress@nhs.net](mailto:emailaddress@nhs.net)**). (Please note: we receive the greatest number of calls on Mondays and at lunchtimes).** We would appreciate it if you could give us as much notice as possible to ensure your appointment can be allocated to another patient. Failing to attend means we are not using our staff effectively and other patients are waiting longer. If you fail to attend this appointment without informing us, you will not be sent a further appointment and will be discharged back to your GP.

We may remind you of this appointment by text message to your mobile phone.

Further information, including travel advice, is also available on our website ([www.Trust.nhs.uk](http://www.Trust.nhs.uk)).

**Our hospitals are involved in developing new treatments and better care. If you would like to take part in a research study, or want to know more about taking part, please speak to the doctor or nurse caring for you. If you are asked to take part in a research study, we will explain it to you in detail. If you decide not to take part, this will not affect your treatment in any way**.
